# Improved Genomic Identification, Clustering, and Serotyping of Shiga Toxin-Producing *Escherichia coli* Using Cluster/Serotype-Specific Gene Markers

**DOI:** 10.3389/fcimb.2021.772574

**Published:** 2022-01-10

**Authors:** Xiaomei Zhang, Michael Payne, Sandeep Kaur, Ruiting Lan

**Affiliations:** School of Biotechnology and Biomolecular Sciences, University of New South Wales, Sydney, NSW, Australia

**Keywords:** STEC O157:H7, non-O157:H7 STEC serotypes, STEC phylogenetic clusters, cluster/serotype-specific gene markers, STEC serotyping, *in silico* STEC tying pipeline STECFinder, metagenomics

## Abstract

Shiga toxin-producing *Escherichia coli* (STEC) have more than 470 serotypes. The well-known STEC O157:H7 serotype is a leading cause of STEC infections in humans. However, the incidence of non-O157:H7 STEC serotypes associated with foodborne outbreaks and human infections has increased in recent years. Current detection and serotyping assays are focusing on O157 and top six (“Big six”) non-O157 STEC serogroups. In this study, we performed phylogenetic analysis of nearly 41,000 publicly available STEC genomes representing 460 different STEC serotypes and identified 19 major and 229 minor STEC clusters. STEC cluster-specific gene markers were then identified through comparative genomic analysis. We further identified serotype-specific gene markers for the top 10 most frequent non-O157:H7 STEC serotypes. The cluster or serotype specific gene markers had 99.54% accuracy and more than 97.25% specificity when tested using 38,534 STEC and 14,216 non-STEC *E. coli* genomes, respectively. In addition, we developed a freely available *in silico* serotyping pipeline named STECFinder that combined these robust gene markers with established *E. coli* serotype specific O and H antigen genes and *stx* genes for accurate identification, cluster determination and serotyping of STEC. STECFinder can assign 99.85% and 99.83% of 38,534 STEC isolates to STEC clusters using assembled genomes and Illumina reads respectively and can simultaneously predict *stx* subtypes and STEC serotypes. Using shotgun metagenomic sequencing reads of STEC spiked food samples from a published study, we demonstrated that STECFinder can detect the spiked STEC serotypes, accurately. The cluster/serotype-specific gene markers could also be adapted for culture independent typing, facilitating rapid STEC typing. STECFinder is available as an installable package (https://github.com/LanLab/STECFinder) and will be useful for *in silico* STEC cluster identification and serotyping using genome data.

## Introduction

Shiga toxin-producing *Escherichia coli* (STEC) are an important cause of foodborne disease worldwide (Tuttle et al., 1999; Teunis et al., 2008; World Health Organization, 2019). STEC causes human infections ranging from mild non-bloody diarrhea to haemorrhagic colitis (HC), haemolytic uraemic syndrome (HUS), end-stage renal disease (ESRD) and death (Paton and Paton, 1998; Tarr et al., 2005; Gould et al., 2009). Globally, an estimated 2.8 million STEC infections resulted in 3,890 cases of HUS, 270 cases of ESRD and 230 deaths in 2010 (Majowicz et al., 2014). Importantly, STEC infections were more frequent and severe in children younger than five years old (Gould et al., 2009; Buvens et al., 2012; Lozer et al., 2013).

Currently, there are over 470 STEC serotypes recognized based on *E. coli* O antigen (determination of O serogroup) and H (flagellar) antigen typing (Gyles, 2007; Mora et al., 2011; Ludwig et al., 2020). More than 130 STEC serotypes are associated with human STEC infections (Johnson et al., 1996; Bettelheim, 2000; Johnson et al., 2006; Valilis et al., 2018). STEC O157:H7 is the most frequent STEC serotype associated with foodborne outbreaks and human infections (Bettelheim, 2000; Qin et al., 2015; Li et al., 2017). However, other non-O157:H7 STEC serotypes have also been a major cause of foodborne outbreaks and sporadic cases, and are responsible for up to 50% STEC infections in recent years (Paton et al., 1999; McCarthy et al., 2001; Paciorek, 2002; Liptáková et al., 2005; Johnson et al., 2006; Zhang et al., 2007; European Food Safety Authority, 2011; Frank et al., 2011a; Käppeli et al., 2011; Verstraete et al., 2013; Zweifel et al., 2013; Morton et al., 2017). Among STEC non-O157:H7 serotypes, six serogroups O26, O45, O103, O111, O121 and O45, also known as “The Big six” (comprising nine serotypes: O26:H11/H-; O45:H2; O103:H2, H11, H25; O111:H8/H-; O121:H19 or H7; and O145:H28/H-) account for over 70% of non-O157:H7 STEC infections (Brooks et al., 2005; Hedican et al., 2009; Bosilevac and Koohmaraie, 2011).

Shiga toxin (Stx) is the main characteristic that defines STEC (Nataro and Kaper, 1998; Tarr et al., 2005), which is encoded by *stx* genes located within lambdoid prophages (Stx-converting phages or Stx-phages) (O'Brien et al., 1989; Mizutani et al., 1999; Bryan et al., 2015; Lacher et al., 2016). Shiga toxins are classified into two types, Stx1 and Stx2. Each of Stx type comprises several subtypes with three subtypes for Stx1 (Stx1a, Stx1c and Stx1d) and 10 subtypes for Stx2 (Stx2a, Stx2b, Stx2c, Stx2d, Stx2e, Stx2f, Stx2g, Stx2h, Stx2i and Stx2k) (Scheutz et al., 2012; Lacher et al., 2016; Bai et al., 2018; Yang et al., 2020). Stx1 and/or Stx2 carrying STEC can cause human disease, however, Stx2 is more often associated with HC and HUS (Lentz et al., 2011; Krüger and Lucchesi, 2015). Among Stx2 subtypes, Stx2a is the most prevalent subtype association with severe disease, followed by Stx2c and Stx2d (Feng and Reddy, 2013; Melton-Celsa, 2014; Krüger and Lucchesi, 2015). *Shigella dystenteriae* and some strains of *Shigella sonnei*, *Shigella flexneri* and *E. albertii* also produce Stx (Beutin et al., 1999; Gupta et al., 2007; Ooka et al., 2012; Gray et al., 2014; Murakami et al., 2014; Brandal et al., 2015). In addition to Shiga toxin, some STEC serotypes also carry the locus of enterocyte effacement (LEE) pathogenicity island (McDaniel and Kaper, 1997; Kaper et al., 2004) responsible for adherence during STEC infections.

STEC serotype detection and identification rely on the detection of Stx proteins by enzyme immune assays or detection of the presence of *stx* genes by molecular methods such as PCR (Brian et al., 1992; Milley and Sekla, 1993; Bélanger et al., 2002; Hara-Kudo et al., 2007; Teel et al., 2007; Zhang et al., 2012). Conventional phenotypic serotyping through antigenic agglutination can further classify STEC to the serotype level (Gyles, 2007). However, cross-reactivity, lack of expression of O antigens, a focus on STEC O157:H7 and novel serotypes may all prevent accurate serotyping and lead to under-detection of non-O157:H7 STEC (Liu et al., 2008; Stigi et al., 2012). Molecular methods, including microarrays, utilising the sequence variations in the O antigen gene clusters, have been developed to serotype STEC O157:H7, “Big six” STEC non-O157:H7 and other STEC serotypes (DebRoy et al., 2004; Gonzales et al., 2011; Lin et al., 2011; Norman et al., 2012; Iguchi et al., 2015; Ludwig et al., 2020). More recently, WGS based methods have been developed for *in silico* serotyping STEC, which allow phenotypically untypeable isolates be serotyped *in silico* using O antigen and flagellin H antigen genes (Inouye et al., 2014; Joensen et al., 2015).

Alongside STEC serotyping which is useful in outbreak investigation and for prevalence surveillance (FAO/WHO STEC EXPERT GROUP, 2019), other subtyping methods such as pulsed-field gel electrophoresis (PFGE), multiple locus variable-number tandem repeat analysis (MLVA) and multilocus sequence typing (MLST) were also used for STEC outbreak investigations (Gerner-Smidt et al., 2006; Gyles, 2007; Frank et al., 2011b). Recently, WGS based typing and metagenomic sequencing have shown great potential for STEC surveillance and outbreak investigation with high resolution and specificity (Leonard et al., 2015; Parsons et al., 2016).

STEC serotypes with the same O and H antigens were generally clustered together and share a common ancestor (Ju et al., 2012). A recent phylogenetic analysis on 276 STEC isolates belonging to 81 serotypes revealed that some STECs formed discrete clades with clustering associated with sequence types and serotypes (González-Escalona and Kase, 2019). Our present study aimed to i), identify phylogenetic clusters of STEC through large scale examination of publicly available genomes; ii), identify cluster/serotype-specific genes for detection of STEC isolates and for detection and serotyping of most frequent STEC serotypes through comparative genomic analysis of accessory genomes; iii), develop an automated pipeline for STEC *in silico* cluster typing and serotyping from WGS data based on cluster/serotype-specific gene markers combined with *E. coli* O and H antigen genes.

## Materials and Methods

### Identification of STEC Isolates From NCBI Database

*E. coli* isolates from the NCBI SRA (National Center for Biotechnology Information Sequence Read Archive) in June of 2020 were queried. The keyword “*Escherichia coli*” was used to retrieve SRA accession numbers of *E. coli* isolates. Raw reads were retrieved from ENA (European Nucleotide Archive). The *stx* genes (*stx_1_*, GenBank accession number M19437; *stx_2_* GenBank accession number X07865) and *ipaH* gene (GenBank accession number M32063) were used to screen *E. coli* reads using Salmon v0.13.0 (Patro et al., 2017). Taxonomic classification for *E. coli* was confirmed by Kraken v1.1.1 (Wood and Salzberg, 2014). Isolates that were positive to any *stx* genes and negative to the *ipaH* gene (the latter to exclude *Shigella* or enteroinvasive *E. coli* [EIEC]) were selected to form the STEC dataset.

A control dataset that represented the sequence types (STs) and ribosomal STs (rSTs) of *stx* negative *E. coli* (“non-STEC”) isolates was constructed. STs and rSTs of non-STEC isolates were obtained from the *E. coli/Shigella* database in Enterobase on August 2020 (Zhou et al., 2020). For STs and rSTs with only one isolate, the isolate was selected. For STs and rSTs with more than one isolate, one representative isolate for each ST and rST were randomly selected. In total, 14,126 *stx*-negative *E. coli* isolates representing 4,354 STs and 11,520 rSTs were selected as non-STEC control database.

### Genome Assembly and Data Processing

Raw reads were assembled *de novo* using SPADES v3.14.0 assembler with default settings [http://bioinf.spbau.ru/spades] (Bankevich et al., 2012). The metrics of assembled genomes were obtained with QUAST v5.0.0 (Gurevich et al., 2013). Three standard deviations (SD) from the mean for contig number, largest contig, total length, GC, N50 and genes were used as quality filter for assembled genomes.

The STs for isolates in the STEC database were checked using mlst (https://github.com/tseemann/mlst) with the *E. coli* scheme from PubMLST (Jolley and Maiden, 2010). rSTs were extracted from the *E. coli/Shigella* rMLST database in Enterobase on August 2020 (Zhou et al., 2020). Serotyping of *E. coli* O and H antigen types were predicted by using SerotypeFinder v2.0.1 (Joensen et al., 2015). The phylogroups of STEC isolates were obtained using ClermonTyping (Beghain et al., 2018).

### Selection of Isolates for STEC Identification Dataset

Representative isolates for each ST, rST and serotype in the STEC dataset were selected to form the identification dataset. For STs, rSTs and serotypes with only one isolate, the isolate was selected. For STs, rSTs and serotypes with more than one isolate, one representative isolate for each ST, rST and serotype was randomly selected. For rSTs in the top six STs, one representative isolate for each rST with two or more isolates was randomly selected. A further 691 isolates including 72 *Escherichia coli* reference (ECOR) strains downloaded from Enterobase, 573 non-STEC *E.coli* isolates representing 573 STs with more than nine genomes, 41 *Shigella* and EIEC isolates representing each cluster identified in our previous study (Zhang et al., 2021), three *E. albertii* isolates and two *E. fergusonii* isolates were used as controls for the identification dataset. The details of the identification dataset are listed in **Table S1**. The remaining STEC isolates in the STEC database were referred to as the validation dataset (**Table S2**).

The identification dataset was used to identify the phylogenetic relationships of STEC isolates and was also used to identify cluster/serotype-specific gene markers. The validation dataset was used to evaluate the performance of cluster/serotype-specific gene markers relative to phylogenetic relationships.

### Phylogeny of STEC Isolates Based on WGS

Phylogenetic trees including an identification tree and 15 validation trees were constructed by using Quicktree v1.3 (Hu et al., 2020) with default parameters to identify and confirm the phylogenetic clustering of STEC isolates. The phylogenetic trees were visualised by Grapetree and ITOL v5 (Zhou et al., 2018; Letunic and Bork, 2019).

The identification phylogenetic tree was generated using isolates in the identification dataset for the identification of clusters of STEC isolates. The validation trees were constructed using isolates in the STEC validation dataset and a subset of isolates known to represent each identified cluster from the identification dataset to assign validation dataset isolates to the clusters defined.

### Identification of the Cluster/Serotype-Specific Gene Markers

Cluster/serotype-specific gene markers were identified from STEC accessory genomes. The genomes from the identification dataset were annotated using PROKKA v1.13.3 (Seemann, 2014). Pan- and core-genomes were analysed by Roary v3.12.0 (Page et al., 2015) using an 80% sequence identity threshold. The candidate gene markers specific to each cluster/serotype were identified from accessory genes with an in-house python script from our previous study (Zhang et al., 2021). The best performing specific gene marker set was selected from the candidates by using BLASTN to search against the identification dataset.

As in our previous studies (Zhang et al., 2019; Zhang et al., 2021) the genomes from a given cluster containing all specific gene markers for that cluster were termed true positives (TP), the genomes from the same cluster lacking any of those same gene markers were termed false negatives (FN). The genomes from other clusters containing all of those same gene markers were termed false positives (FP). The sensitivity (True positive rate, TPR) of each cluster-specific gene marker was defined as TP/(TP+FN). The specificity (True negative rate, TNR) was defined as TN/(TN+FP).

### Validation of the Cluster/Serotype-Specific Gene Markers

The specific gene markers were examined by using BLASTN to search against the validation dataset (**Table S2**) and non-STEC *E. coli* control database for the presence of any of the cluster/serotype-specific gene markers. The BLASTN thresholds were defined as 80% sequence identity and 50% gene length coverage.

### Development of STECFinder, an Automated Pipeline for Molecular Serotyping of STEC

**Figure 1:**
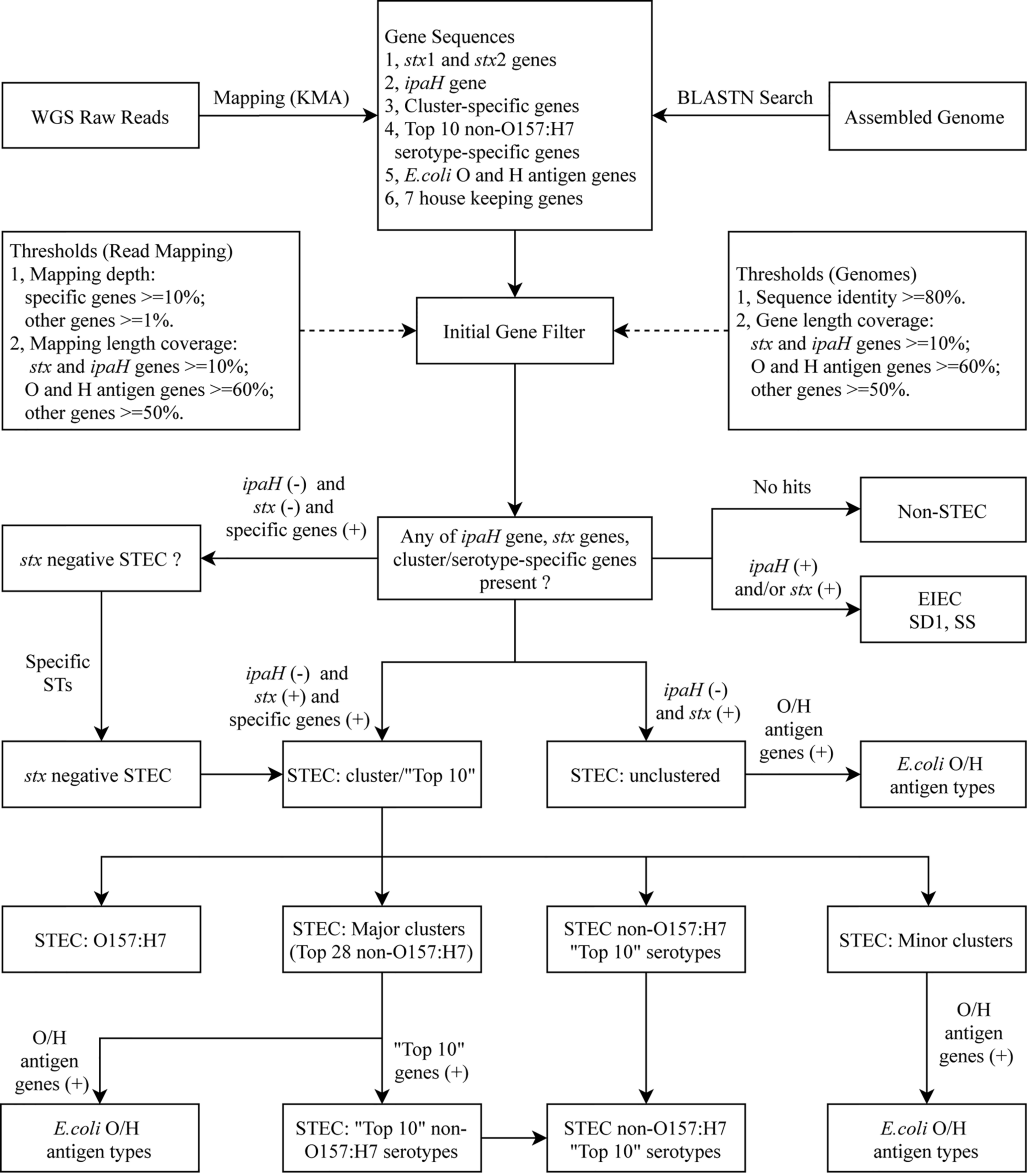
*in silico* serotyping pipeline workflow. Schematic of *in silico* serotyping STEC by cluster/serotype-specific genes combined with the *ipaH* gene, *stx* genes including all available subtypes and *E. coli* O antigen and H antigen genes, implemented in STECFinder. Both assembled genomes and raw reads are accepted as data input.

STECFinder was developed for STEC serotyping from either paired end Illumina genome sequencing reads or assembled genomes. The typing reference sequences used for construction of STECFinder included specific gene marker sets identified in this study, established *E. coli* O antigen and H antigen gene sequences collected from SerotypeFinder (Joensen et al., 2015), *stx* subtype sequences collected from VirulenceFinder and three other studies (Joensen et al., 2014; Lacher et al., 2016; Bai et al., 2018; Yang et al., 2020), the *ipaH* gene sequence downloaded from NCBI, and seven House Keeping (HK) genes -*recA*, *purA*, *mdh*, *icd*, *gyrB*, *fumC* and *adk* from the *E. coli* MLST scheme (Jolley and Maiden, 2010) for contamination checking (**Figure 1**). All sequences are available in fasta format at https://github.com/LanLab/STECFinder with cluster specific genes named with the following convention: STEC-cluster-gene_number (i.e. STEC-C1-gene_1 for the first gene in the C1 specific set).

For the analysis of sequence data as raw reads, KMA (*k*-mer alignment) v1.3.15 (Clausen et al., 2018) was used to align the raw reads to the typing reference sequences. KMA utilizes *k*-mer seeding and the Needleman-Wunsch algorithm (Needleman and Wunsch, 1970) to accurately align reads to genes of interest. The best-aligning template was chosen from a novel sorting scheme ConClave scheme incorporated into KMA (Clausen et al., 2018). To determine whether the genes were present or absent, the mapping length coverage and a minimum depth were used as the thresholds.

For the submission of sequence data as assembled genomes, BLASTN v2.9.0 (Camacho et al., 2009) was used to search against the typing reference sequences with 80% sequence identity. The presence or absence of genes was determined by the gene length coverage.

The presence or absence of genes in STECFinder was determined by the cutoff value of gene length coverage for assembled genomes and the mapping length coverage and a minimum mapping depth for raw reads. For assembled genomes, length coverage of 50% for all cluster/serotype-specific genes, 60% for O and H antigen genes and 10% for *ipaH* gene and *stx* genes were used as cutoff value for determination of the presence of genes. For raw reads, mapping length coverage of 50% for all cluster/serotype-specific genes, 60% for O and H antigen genes, 10% for *ipaH* gene and *stx* genes and a minimum depth of 10 for all cluster-specific genes, a minimum depth of one for O and H antigen genes, *ipaH* gene and *stx* genes were used to define the gene as present. In addition, when multiple O and H genes were detected the bitscore was incorporated into STECFinder for filtering and ranking O and H antigen. The highest match was chosen as the O or H antigen present, when multiple O or H variants were present.

The major and minor clusters and top 10 non-O157:H7 STEC serotypes were assigned based on the presence of cluster/serotype-specific gene marker set together with the presence of *stx* subtypes and the absence of *ipaH* gene. All genes in a cluster/serotype-specific gene set must be defined as present for a cluster or serotype to be called. An ‘unclustered’ was assigned for isolate that cannot be detected by any of cluster-specific gene marker set. Unclustered STEC could be new clusters or isolates that contained all genes in the marker set but one or more genes from marker set did not pass the cutoff value.

Additional subsets of gene marker sets were added to increase the accuracy of clusters and calling of the top 10 non-O157:H7 STEC serotypes. For example, the combination of the specific gene marker set of O157:H7 and AM18 can eliminate the known false presences of AM18 gene set in O157:H7. The isolate is assigned as AM18 if both gene sets are present while the isolate is assigned as O157:H7 if AM18 specific gene set is absent. The subsets of combined gene sets were incorporated into the STECFinder for elimination of false cluster assignment are listed in **Table S6**.

STECFinder was tested with identification dataset. The accuracy and specificity of STECFinder for prediction of clusters and serotypes were evaluated with STEC validation dataset and non-STEC *E.coli* control dataset.

### Application of STECFinder in STEC Typing Using Metagenomics Data From STEC Spiked Food Samples

STECFinder can take input from metagenomics sequencing reads for STEC typing. The application of STECFinder in metagenomics analysis was evaluated using 17 metagenomic sequencing read sets from samples published by Buytaers et al. (Buytaers et al., 2020). These 17 shotgun metagenomic sequencing reads (Buytaers et al., 2020) were downloaded from ENA.

## Results

### Screening Sequenced Genomes for STEC Isolates

The presence of any of *stx* genes and the absence of the *ipaH* gene were used to identify STEC isolates. We examined 140,348 isolates with the species annotation of *E. coli* with paired end Illumina sequencing reads available in ENA database. Of the 140,348 isolates, 43,960 isolates were positive to *stx_1_* and/or *stx_2_* genes and negative for the *ipaH* gene. 41,101 of the 43,960 isolates passed taxonomic classification and genome assembly quality filters and were selected to form the STEC dataset.

Isolates in the STEC dataset were typed using MLST, rMLST and SerotypeFinder. MLST typed the 41,101 STEC isolates into 817 STs (202 isolates not typed by MLST) of which 368 STs were represented by a single isolate, 424 STs represented by two to 100 isolates each and accounted for 12% of the STEC isolates, whereas 25 STs contained more than 100 isolates each and encompassed 86.61% of the STEC isolates, of which ST11 is the largest, accounting for 37.12% of the STEC isolates, followed by ST21 (14.71%), ST17 (11.91%), ST16 (6.72%), ST655 (2,71%) and ST32 (2.46%). rMLST divided the 41,101 STEC isolates into 2,911 rSTs (12,208 isolates not typed by rMLST).

Using SerotypeFinder, 38,958 of the 41,101 (94.79%) isolates were assigned to 460 *E. coli* O:H antigen types, 2,039 isolates (4.96%) were not assigned an O antigen and were typed for H antigens only with 38 H antigen types, of which H7, H2, H8, H11 and H21 were the most frequent types, 96 isolates (0.23%) were typed as multiple O:H types and six isolates (0.01%) were untypeable.

### The Frequency of STEC Serotypes

The 38,958 STEC O:H antigen typeable isolates belonged to 460 different serotypes including O157:H7 (38.55% of 38,958 typeable isolates) and 459 non-O157:H7 serotypes (61.45% of 38,958 typeable isolates).

**Figure 2:**
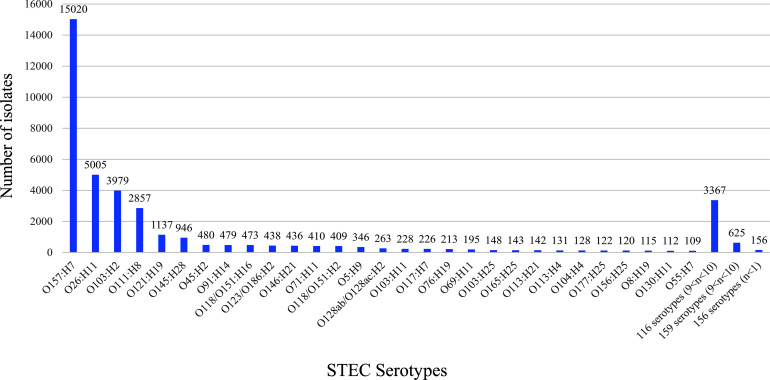
The frequency of 460 STEC serotypes. The graph shows the frequency of 460 STEC serotypes. STEC O157:H7 and top 28 non-O157:H7 serotypes are listed separately. The number on top of each stacked column refers to the number of isolates for each serotype.

Of the 459 non-O157:H7 serotypes, the top 28 serotypes were present in more than 100 isolates each and accounted for 50.8% of 38,958 typeable STEC isolates, of which the 10 most frequent serotypes (41.66% of 38,958 typeable STEC isolates) were O26:H11, O103:H2, O111:H8, O121:H9, O145:H28, O45:H2, O91:H14, O118/O151:H16, O123/O186:H2 and O146:H21. The top 6 serotypes belonged to the well-known “Big six” STEC non-O157 serogroups (Brooks et al., 2005; Hedican et al., 2009; Bosilevac and Koohmaraie, 2011). It should be noted that three serotypes, O103:H11, O103:H25, and O121:H7, belonging to the “Big six” non-O157 STEC serogroups were outside the top 10 serotypes. The 116 serotypes present with 10 to 100 isolates each, belonged to 8.64% of typeable STEC isolates. The remaining 315 serotypes with less than 10 isolates each represented 2% of the typeable STEC isolates (**Figure 2**).

### Identification of STEC Clusters

**Figure 3:**
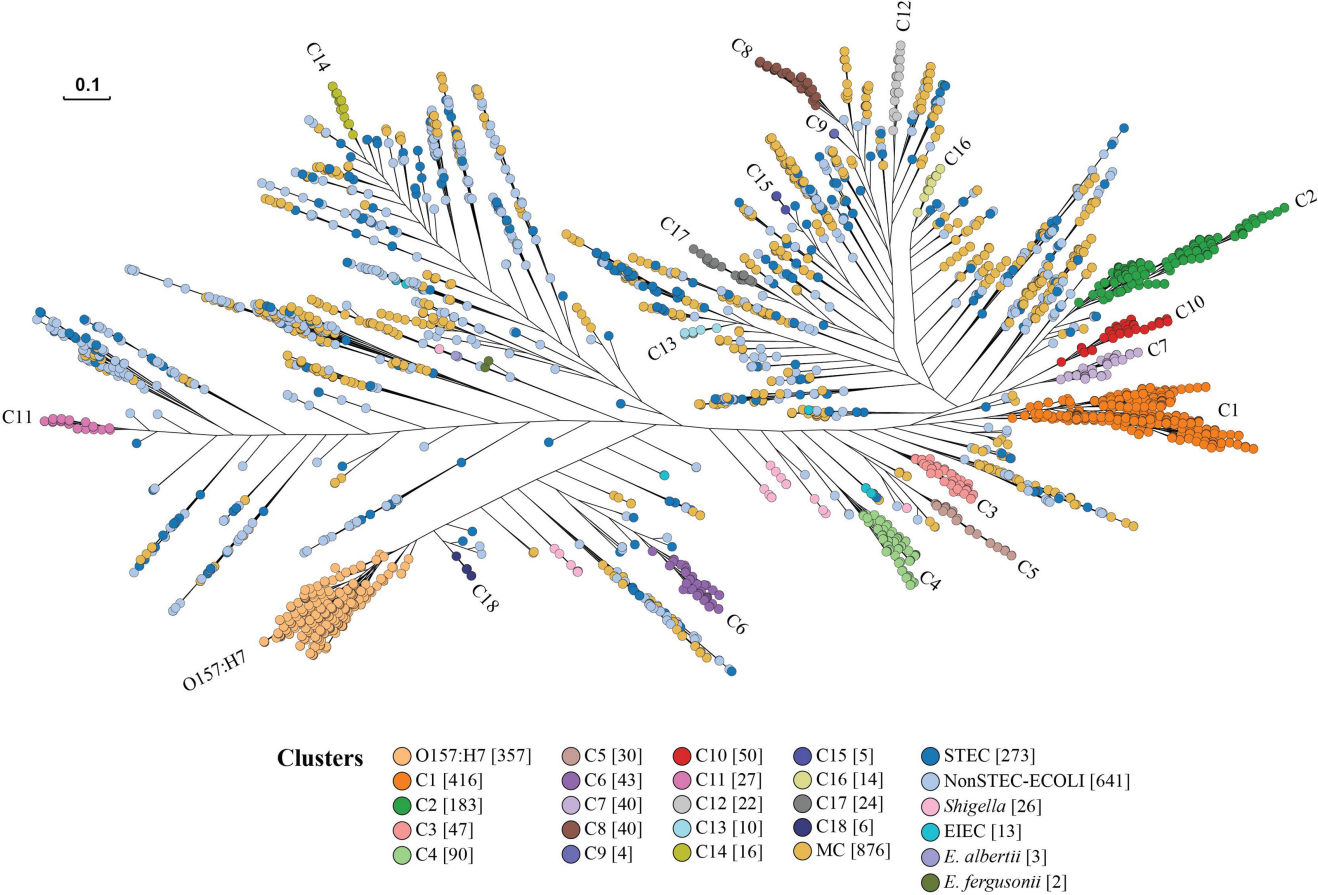
STEC cluster identification phylogenetic tree. Representative isolates from the identification dataset were used to construct the phylogenetic tree by Quicktree v1.3 to identify STEC (Shiga toxin-producing *E. coli*) clusters and visualised using Grapetree. The dendrogram shows the phylogenetic relationships of 2,567 STEC isolates represented in the identification dataset. Branch lengths are log scale for clarity. The scale bar represents 0.1 substitutions per site. STEC clusters are coloured. Numbers in square brackets after cluster name are the number of isolates for each identified cluster. ECOLI is *E. coli.* EIEC is Enteroinvasive *E. coli*. MC indicates a minor STEC cluster.

To identify any phylogenetic clusters containing one or more STEC serotypes from the 41,101 STEC isolates, we selected representative isolates to perform phylogenetic analysis as it was impractical to construct a tree with all isolates. The selection was performed on the basis of ST, rST and serotype of the 41,101 STEC isolates. One isolate was selected to represent each ST, rST and serotype for a total of 2,567 STEC isolates. Note that in the case that STs or rSTs overlapped with serotype, an isolate was only selected once to avoid duplicates of the same isolate. The selection included 817 STs, 1,413 rSTs, 460 STEC serotypes and 102 partial antigen types (H antigen only and multiple O/H types). A further 691 isolates consisting of 72 ECOR isolates, 573 non-STEC *E.coli* isolates, 41 *Shigella* and EIEC isolates, three *E. albertii* isolates and two *E. fergusonii* isolates were also included. The identification dataset consisted of 3,258 isolates in total. Details are listed in **Table S1**. A phylogenetic tree was constructed using 3,258 isolates in the identification dataset to identify the clusters (**Figure 3**).

The identification of clusters was focused on O157:H7 and the top 28 non-O157:H7 serotypes. A major cluster was defined if the cluster node had a bootstrap value of above 80% and contained STEC isolates belonging to O157:H7 and top 28 non-O157:H7 serotypes. The isolates of O157:H7 were grouped into one large cluster. A further 18 major clusters (C1-C18) all of which carried only non-O157:H7 serotypes (**Figure 3**; **Table 1** and **Figure S1**), were identified. The isolates of top 28 non-O157:H7 serotypes fell into these 18 major clusters. Of the 2,567 STEC isolates, 1,412 fell within the O157:H7 cluster or one of the 18 non-O157:H7 major STEC clusters.

**Table 1:** Major STEC clusters identified in identification dataset.

Cluster	No. of isolates	No. of serotypes	No. of STs	Top 28 non-O157:H7 serotypes*
O157:H7	356	1	83	O157:H7
C1	414	30	97	1-O26:H11, 3-O111:H8, 8-O118/O151:H16,
				12-O71:H11, 15-O103:H11, 18-O69:H11
C2	181	16	42	2-O103:H2, 6-O45:H2, 9-O123/O186:H2
				11-O118/O151:H2
C3	45	18	12	19-O103:H25, 25-O156:H25, 6-O45:H2
C4	89	14	21	13-O5:H9, 20-O165:H25, 24-O177:H25
C5	29	1	5	4-O121:H19
C6	41	1	6	5-O145:H28
C7	40	2	13	7-O91:H14
C8	40	1	14	10-O146:H21
C9	4	1	1	10-O146:H21
C10	50	2	15	14-O128ab:H2
C11	27	1	6	16-O117:H7
C12	21	1	6	17-O76:H19
C13	10	1	7	21-O113:H21
C14	16	2	2	22-O113:H4
C15	5	1	1	23-O104:H4
C16	14	1	4	26-O8:H19
C17	24	11	7	27-O130:H11
C18	6	1	1	28-O55:H7

*The serotypes in each non-O157:H7 cluster are listed with their rank by isolate frequency for the top 28 non-O157:H7 serotypes followed by the serotype.

Of the remaining 1,155 STEC isolates, 877 isolates were grouped into 229 STEC minor clusters with two or more isolates in a cluster, whereas 278 isolates were singletons separated from other clusters by non-STEC *E. coli* isolates. We further typed the isolates from minor clusters using phylogroup typing (Brooks et al., 2005) and each minor cluster was named by phylogroup and lineage number, for example, phylogroup A minor cluster 1 (AM1). Most of the minor clusters belonged to phylogroup B1 (**Table 2**).

**Table 2:** Summary of identified STEC minor clusters in identification dataset.

Phylogroup	No. of MC*	Name of MC	No. of isolates	No. of serotypes	No. of STs
A	37	AM1-AM37	139	64	42
B1	126	B1M1-B1M126	519	157	186
B2	14	B2M1-B2M14	35	20	17
C	7	CM1-CM7	17	10	8
D	22	DM1-DM22	67	26	29
E	19	EM1-EM19	73	26	34
G	4	GM1-GM4	27	12	12

*MC, minor clusters.

**Figure 4:**
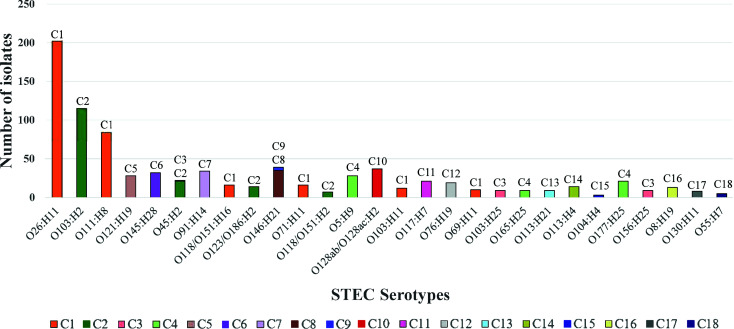
The frequency of the top 28 non-O157:H7 STEC serotypes in STEC major clusters. The graph shows the frequency of top 28 non-O157:H7 serotypes in the 18 STEC major clusters. Clusters are shown per colour legend and also at the top of the bar. X-axis shows the serotype while y-axis shows the number of isolates.

In total, 19 major STEC clusters including one O157:H7 and 18 non-O157:H7 clusters (Top 28 non-O157:H7 serotypes) and 229 STEC minor clusters containing other non-O157:H7 serotypes were identified. Of the 19 major clusters, 12 had a single serotype and seven had two or more serotypes. The frequency of non-O157:H7 STEC serotypes in the major clusters are shown in **Figure 4**. For the 229 STEC minor clusters, 103 contained a single serotype, 109 consisted of two or more serotypes and the remaining 17 comprised of isolates with H antigen types only.

Among the top 10 non-O157:H7 serotypes, O121:H19 (C5), O145:H28 (C6), O91:H14 (C7) had a single origin while O146:H21 (C8 and C9) was a paraphyletic serotype. O26:H11 and O118/O151:H16 were grouped into C1. O123/O186:H2 was grouped into C2. O103:H2, O111:H8 and O45:H2 had polyphyletic origins. O103:H2 and O111:H8 were grouped into C2 and B1M118, C1 and B1M119, respectively. O45:H2 had three lineages which were clustered into C2, C3 and AM37. Three serotypes (O128ac:H2, O8:H19 and O113:H21) of the remaining top 28 non-O157:H7 serotypes were polyphyletic serotypes. Thirty non top 28 non-O157:H7 serotypes also had polyphyletic origins.

Apart from STEC isolates, 26 of the 573 *stx* negative *E. coli* isolates from the identification dataset were grouped into clusters. Of the 19 major clusters identified, 12 contained *stx* negative *E. coli* isolates (ST11 in O157:H7; ST765 and ST29 in C1; ST17, and ST376 in C2; ST343 andST300 in C3, ST342 in C4; ST655 in C5; ST32 in C6; ST442 and ST1992 in C8, ST335 in C18). These STs containing *stx* negative *E. coli* isolates were the most frequent STs in the STEC database, suggesting these *stx* negative *E. coli* isolates may have lost the *stx* genes. The details of STEC clusters and lineages were listed in **Table S3**.

However, 11 STEC minor clusters also contained *stx* negative *E. coli* isolates. Therefore, we further examined STs with more than two isolates from all minor STEC clusters that were also found within the 14,126 *stx* negative *E. coli* (“non-STEC”) isolates. Of the 229 minor STEC clusters, the STs in 58 clusters contained *stx* positive isolates only and the STs in 171 clusters contained both *stx* negative and *stx* positive isolates. Of these 171 minor STEC clusters, the STs in four clusters consisted of *stx* positive isolates and *E. coli* isolates that didn’t carry typical pathotype specific genes (data not shown). While STs in the remaining 167 clusters consisted of *stx* positive isolates and isolates that carried pathotype specific genes from other *E. coli* pathotypes (data not shown). Thus, these STEC minor clusters are a mix of STEC and other pathotypes.

### Identification of the Cluster/Serotype-Specific Gene Markers

In this study, we used the same definition and approach as used to find the *Shigella*/EIEC cluster specific genes (Zhang et al., 2021). We searched for potential specific gene marker sets for the 19 major and 229 minor clusters using the accessory genomes from the 3,258 identification dataset isolates. Genes associated with STEC O antigen gene clusters were excluded from the analysis to identify O antigen gene independent markers. Multiple candidate cluster/serotype-specific gene marker sets for each of the 19 major STEC clusters and 229 minor STEC clusters were identified. The single gene marker set with 100% sensitive and the highest specificity were then selected from candidate cluster-specific gene marker sets by BLASTN searches against genomes in the identification dataset using 80% sequence identity and 50% gene length threshold.

We also searched for specific gene markers for six of the top 10 non-O157:H7 serotypes (O26:H11, O111:H8, O118/O151:H16, O103:H2, O45:H2 and O123/O186:H2) which were not in a cluster of their own. The best performing gene marker set for each of six of top 10 non-O157:H7 serotypes were identified using the same approach as used to identify and select cluster-specific gene marker sets.

The sensitivity and specificity of each major STEC cluster and six non-O157:H7 serotype specific gene marker set for the identification dataset were listed in **Table 3**. The major STEC cluster and six non-O157:H7 serotype specific gene marker sets were all 100% sensitive and the specificity varied from 99.72% to 100% for major STEC cluster-specific gene marker sets and from 99.41% to 100% for non-O157:H7 serotype-specific gene marker sets. The STEC minor cluster-specific gene marker sets were 100% specific with the exception of 12 minor clusters which had specificity ranging from 99.85% to 99.97% (**Table S4**).

**Table 3:** The sensitivity and specificity of STEC cluster/serotype-specific gene markers.

Clusters	Cluster-specific genes marker sets	Identification dataset (3,258 isolates)		
		No. of isolates	Sensitivity	Specificity*
O157:H7	Set of 6 genes	356	100	99.72
C1	Set of 4 genes	414	100	99.82
C2	Set of 4 genes	181	100	99.97
C3	Set of 3 genes	45	100	100
C4	Set of 3 genes	89	100	99.97
C5	Set of 4 genes	29	100	100
C6	Set of 3 genes	41	100	99.88
C7	Set of 4 genes	40	100	99.97
C8	Set of 5 genes	40	100	99.97
C9	Set of 2 genes	4	100	100
C10	Set of 2 genes	50	100	100
C11	Single gene	27	100	100
C12	Set of 2 genes	21	100	100
C13	Set of 4 genes	10	100	100
C14	Set of 4 genes	16	100	99.97
C15	Set of 2 genes	5	100	100
C16	Set of 4 genes	14	100	99.97
C17	Set of 3 genes	24	100	99.97
C18	Set of 3 genes	6	100	99.97
O26:H11	Set of 6 genes	204	100	99.41
O103:H2	Set of 4 genes	121	100	99.87
O111:H8	Set of 3 genes	96	100	100
O45:H2 (C2)	Set of 5 genes	22	100	99.97
O45:H2 (C3)	Set of 3 genes	1	100	100
O118/O156:H16	Set of 4 genes	17	100	99.94
O123/O186:H2	Set of 3 genes	21	100	100

^*^The specificity of cluster-specific gene set less than 100% was due to at least one false positive found in that set.

### Validation of Cluster/Serotype-Specific Gene Markers

The STEC cluster/serotype-specific gene marker sets were evaluated with 38,534 STEC isolates from the validation dataset and 14,126 isolates from non-STEC *E.coli* control dataset.

The STEC cluster-specific gene marker sets were able to assign 35,464 of 38,534 (92.03%) STEC isolates to the major clusters and 2,703 (7.01%) STEC isolates to minor clusters. In total, 38,155 of 38,534 (99.02%) STEC isolates can be assigned to clusters by cluster-specific gene marker sets, while 150 of the 38,534 (0.39%) STEC isolates were assigned with more than one cluster and 217 of the 38,534 (0.56%) STEC isolates were not assigned to any cluster by STEC cluster-specific gene marker sets.

Validation phylogenetic trees (**Figure S2**) were then constructed to confirm the assignment of cluster-specific gene marker sets. We divided the 38,534 STEC validation isolates into 15 subgroups. Each of the 15 subgroups isolates together with a subset of 476 STEC isolates with known clusters and 691 non-STEC isolates from identification dataset were used to generate validation trees for a total of 15 validation trees. The validation isolates were considered to truly belong to a given cluster if the isolates were found within a branch that only contained identification dataset isolates from that cluster with a bootstrap value of 80% or greater. In total 38,340 (99.5%) validation isolates were assigned to major and minor STEC clusters with 35,574 (92.32%) and 2,766 (7.18%) respectively, while the remaining 194 isolates (0.5%) were not assigned to any clusters.

Compared to cluster assignment by phylogenetic trees as the ground truth, cluster-specific gene marker sets correctly assigned 35,461 validation isolates to major clusters and 2,704 validation isolates to minor clusters. Cluster -specific gene marker sets also correctly identified 191 of the 194 isolates without cluster assignments. In total the accuracy of assignments by cluster -specific gene marker sets were 99.54%. The sensitivity and specificity for each cluster-specific gene marker set for validation dataset were listed in **Table S4**.

The STEC cluster specific gene marker sets were validated on 14,216 non-STEC *E. coli* isolates. The specificity of the STEC cluster-specific gene markers set for major clusters varied from 99.38% to 100% and the specificity of the STEC cluster-specific gene marker sets for minor clusters ranged from 97.25% to 100%. Details are listed in **Table S5**.

### STECFinder for Molecular Serotyping of STEC Isolates and Its Accuracy and Specificity

STECFinder was developed for cluster and serotype identification of STEC isolates. Cluster was identified using cluster -specific gene marker sets and serotype was identified using serotype-specific gene markers as well as *E. coli* O and H antigen genes within clusters. Either paired end Illumina genome sequencing reads or assembled genomes can be used. STECFinder is available on github (https://github.com/LanLab/STECFinder).

The accuracy and specificity of STECFinder for STEC typing were tested with 3,258 isolates from the identification dataset. For assembled genomes, all 1,412 STEC isolates belonging to 19 major clusters and all 877 STEC isolates belonging to 229 minor clusters were correctly predicted, while 26 of 573 *stx* negative *E.coli* isolates were assigned to STEC clusters by their corresponding cluster-specific gene marker sets. Eighteen STEC singletons were assigned to clusters or minor clusters. For read mapping, two of 1,412 isolates belonging to the 19 major clusters and 25 of 877 isolates from minor clusters were not detected by cluster-specific gene marker sets, while 26 *stx* negative *E.coli* was assigned to STEC clusters similar to the assignment using the assembled genomes. The accuracy of STECFinder for cluster assignments was 99.45% and 98.5% for assembled genomes and read mapping respectively. The accuracy of cluster assignment for the top 10 non-O157:H7 serotypes was 99.14% and 99.11% for assembled genomes and read mapping, respectively.

STECFinder was validated on 38,534 isolates from the STEC validation dataset. Compared to the ground truth assignments determined using phylogenetic analysis, STECFinder assigned 99.85% and 99.83% of validation isolates correctly to clusters for assembled genomes and read mapping, respectively. The accuracy of cluster assignment for top 10 non-O157:H7 serotypes was 99.72% for assembled genomes and 99.65% for read mapping. For the 38,534 *stx*-positive isolates from validation dataset, STECFinder demonstrated 100% cluster assignment specificity for both assembled genomes and read mapping. The cluster assignment specificity of STECFinder was further evaluated using the 14,126 *stx*-negative *E. coli* isolates from the “non-STEC” control dataset. The specificity was 87.07% and 85.12% for assembled genomes and read mapping, respectively. Further investigation of the false positive isolates found that 1,074 false positive isolates belonged to the STEC cluster based on phylogenetic analysis. After removing all of these false positive isolates, the specificity was 94.66% and 92.72% for assembled genomes and read mapping respectively.

STECFinder can assign STEC isolates to serotype level within predicted clusters. The comparison of *in silico* serotyping of the total of 41,101 STEC isolates between STECFinder and SerotypeFinder (Joensen et al., 2015) was performed. For assembled genomes, the serotype prediction of 40,912 of 41,101 (99.54%) STEC isolates by STECFinder agreed with that by SerotypeFinder when applying the same cutoff values of 80% sequence identity and 60% length coverage. For the remaining 189 STEC isolates with non-identical serotype prediction, STECFinder predicted serotypes were largely a subset of O:H types predicted by SerotypeFinder. For example, an isolate may be assigned as wzx_O103 and H2 by STECFinder while SerotypeFinder predicted as a mixed wzx_O103/O26 and H2/H11.

There were 40,618 of 41,101 (98.82%) STEC isolates with the same serotype prediction by STECFinder and SerotypeFinder from read mapping. For the remaining 483 cases, STECfinder assigned a full serotype while SerotypeFinder assigned 257 isolates with H antigen only, 117 isolates with O antigen only and 109 isolates with multiple O:H types.

### Detection of STEC Clusters and Serotypes Using STECFinder in Spiked Food Samples Using Shotgun Metagenomic Sequencing Reads

The application of STECFinder in metagenomics analysis was evaluated with 17 metagenomic sequencing reads from samples published by Buytaers et al. (Buytaers et al., 2020). The 17 metagenomic samples consisted of nine minced beef meat samples spiked with a STEC O157:H7 isolate, one fresh goat cheese sample each spiked with STEC O145:H28 isolate, O103:H2 isolate and co-spiked with STEC O103:H2 and O145:H28 isolates and five STEC negative control food samples. Samples were spiked with STEC isolates at the lowest infectious dose (<10 CFU for 25 g of food) (Buytaers et al., 2020).

STECFinder assigned the nine samples spiked with STEC O157:H7 to O157:H7 cluster, one sample with STEC O145:H28 to C6 (O145:H8), one sample spiked with STEC O103:H2 to C2 and O103:H2 (O103:H2 is within C2). One sample co-spiked with STEC O103:H2 and O145:H28 was assigned to C2 and O103:H2 (O103:H2 is within C2), and C6 (O145:H8). The cluster/serotype-specific gene marker sets were not detected in the five control samples and STECFinder assigned the five sequenced reads of STEC negative control to “Other-*E. coli*”. STECFinder correctly typed the spiked samples using cluster/serotype-specific gene markers.

## Discussion

In this study, we performed genomic analysis of more than 41,000 STEC genomes representing 460 different serotypes and identified 19 major phylogenetic clusters including one O157:H7 cluster and 18 non-O157:H7 clusters containing the 28 most frequent non-O157:H7 serotypes, and 229 minor clusters. WGS-based phylogenetic analysis of such a large set of genome data found that STEC had far greater genetic diversity than what has been observed previously with clusters containing one or more serotypes. The close phylogenetic relationship between O26:H11, O111:H8 and O103:H11 in C1, O103:H2 and O45:H2 in C2 agreed with previous studies (González-Escalona and Kase, 2019; Zhang et al., 2020). With the large number of serotypes (460) as well as polyphyletic or paraphyletic origin of 37 serotypes, identification of serotype specific markers for all serotypes was not possible. However, cluster specific markers were identified and used to develop a pipeline, STECFinder, to facilitate cluster and serotype identification of STEC isolates.

STEC infections have a significant impact on public health worldwide (FAO/WHO STEC EXPERT GROUP, 2019). Early detection and differentiation of STEC is vital for food safety surveillance and public health. The initial screening of *stx* genes for STEC serotype detection may lead to misdiagnosis of STEC because *stx* genes can be lost or gained (FAO/WHO STEC EXPERT GROUP, 2019). We identified a small number of *stx*-negative *E. coli* isolates that were grouped into STEC clusters with the corresponding STEC serotypes and STs. Whether these *stx*-negative *E.coli* isolates lost *stx*-containing prophages or were the progenitors of STEC remains unknown. It may also be possible that only a subset of isolates within those STs were *stx* positive due to recent acquisition of *stx*. However, human infections caused by *stx*-negative isolates with typical STEC serotypes have been reported previously (Bielaszewska et al., 2007; Mora et al., 2012; Ferdous et al., 2015). STECFinder will predict these typical STEC serotypes based on cluster/serotype-specific gene markers even if *stx* is absent. It should be noted that STECFinder does not make determination whether a given isolate is an STEC as its key utility is to predict predefined STEC clusters and serotypes. The presence and identity of *stx* genes is also reported to allow the user to make their own determination.

Our analysis found some minor clusters as well as STs contain both *stx* negative and *stx* positive isolates with *stx* negative isolates being of other *E. coli* pathotypes, which suggests that the STEC within those clusters and STs may be hybrid pathogens. Such hybrids have been recognised in recent years including the well-known STEC/EAEC (enteroaggregative *E. coli*) hybrid O104:H4 (ST678) and STEC/UPEC (uropathogenic *E. coli*) hybrid O2:H6 (ST141) (Navarro-Garcia, 2014; Gati et al., 2019). Therefore, for STEC clusters, serotypes or STs that carry isolates with different pathogenicity, a note of caution on the use of STECFinder is required as such clusters identified may not uniquely contain STEC pathogens. More data are needed to determine how many serotypes or STs carry different pathotypes and STECFinder does not attempt to determine other or hybrid pathogenic types. Determining whether an isolate is a hybrid pathogen is often difficult as some pathogenic types were not well defined by the presence of virulence genes.

Serotyping provides valuable information on identification of potential pathogenic STEC (Gyles, 2007; World Health Organization, 2019). Current serotyping methods focus on well-known O157 and “Big six” non-O157 serogroups which account for about 70% of STEC infections. There are many challenges for the detection of other non-O157:H7 serotypes which cause the remaining 30% of STEC infections (DebRoy et al., 2011; Norman et al., 2012; Zweifel et al., 2013; Smith et al., 2014). In addition, not all STEC can be serotyped *in silico* or predicted based on O or H type genes from genome sequencing data (Joensen et al., 2015; González-Escalona and Kase, 2019). STECFinder can accurately predict STEC serotypes including those most frequently associated with foodborne outbreaks and severe disease. STECFinder can also accurately predict other non-”Big six” non-O157:H7 STEC serotypes. This could be beneficial for identification of the most frequent STEC serotypes for early diagnosis and for clinical management and will better inform the genomic surveillance of STEC serotypes.

We verified the serotype of STEC isolates predicted using STECFinder by phylogenetic cluster assignment and shared STs with STEC isolates of known serotypes. Compared with the existing pipeline for *E. coli in silico* serotyping, SerotypeFinder (Joensen et al., 2015), cluster/serotype-specific gene markers based STECFinder can eliminate the majority of uncertain antigen type calls and provides more accurate STEC serotyping within predicted clusters. STECFinder will be useful for epidemiological and diagnostic investigations as well as providing an alternative *in silico* STEC typing method.

We were unable to validate 43 of the 229 minor cluster-specific gene marker sets as these minor clusters had few isolates and once isolates were included in the identification dataset, no isolates remained for validation. Therefore, markers for these 43 minor clusters are tentative and require future validation when more genomes become available. Genes specific to each of these STEC minor clusters were also based on very small number of genomes and should be used with caution. However, since these minor clusters are rarely isolated, they have relatively little effect on the overall applicability of the cluster-specific gene marker sets to STEC typing.

Culture-independent approaches such as shotgun metagenomic analysis may be used for detection of contaminating STEC directly from food samples or enriched food samples (Leonard et al., 2015; Buytaers et al., 2020). However, it is difficult to determine STEC serotype from food or faecal samples directly as O and H antigen genes cannot uniquely identify a STEC serotype in a mixed sample. We showed that the cluster/serotype-specific gene marker sets of interest were detected in the spiked food samples by STECfinder using shotgun metagenomic sequencing reads from the study of Buytaers et al. (2020). Our cluster or serotype specific genes provide proxy markers to identify these serotypes in original or non-pure culture samples. These gene markers could be adapted for metagenomics based diagnosis and culture independent typing, facilitating rapid identification of known STEC clusters and serotypes.

## Conclusion

This study analysed 41,101 publicly available genomes of STEC isolates and identified 19 major and 229 minor STEC clusters. Specific gene marker sets for the 19 major and 229 minor clusters were identified and found to be valuable for *in silico* typing. We also identified serotype specific markers for the top 10 non-O157:H7 STEC serotypes. These markers can be used as proxy markers to identify the serotypes. We additionally developed STECFinder, a freely available *in silico* serotyping pipeline incorporating the cluster/serotype specific gene markers to facilitate serotyping of STEC isolates using genome sequences with high specificity and sensitivity. The STECFinder pipeline was tested on published metagenomics samples to determine the serotype of known STECs and the results show that cluster and serotype specific markers have potential for culture independent STEC serotyping.

## Data Availability Statement

The original contributions presented in the study are included in the article/**Supplementary Material**. Further inquiries can be directed to the corresponding author.

## Author Contributions

RL designed the study. XZ, MP, and SK performed the bioinformatic analysis. XZ, MP, and RL analysed the results. XZ drafted the manuscript. MP and RL provided critical revision of the manuscript. All authors contributed to the article and approved the submitted version.

## Conflict of Interest

The authors declare that the research was conducted in the absence of any commercial or financial relationships that could be construed as a potential conflict of interest.

## Publisher’s Note

All claims expressed in this article are solely those of the authors and do not necessarily represent those of their affiliated organizations, or those of the publisher, the editors and the reviewers. Any product that may be evaluated in this article, or claim that may be made by its manufacturer, is not guaranteed or endorsed by the publisher.

## Supplementary Material

Supplementary Figure S1 Identification phylogenetic tree. The identification phylogenetic tree was constructed using Quicktree v1.3 as **Figure 3** and was visualised using ITOL v5 which allowed bootstrap values to be displayed by colouring the internal nodes. The scalar bar represents 0.01 substitutions per site. STEC (Shiga toxin producing *E. coli*) clusters are colored per cluster legend and shown as the ring. The internal branches are colored to represent the bootstrap values per colour legend with green and red indicating the maximum (1) and minimum bootstrap values (0). Each cluster is supported by bootstrap value of 80% or greater. ECOLI is *E. coli*. EIEC is Enteroinvasive *E. coli*. MC is STEC minor clusters.

Supplementary Figure S2 The representative validation phylogenetic tree.

Supplementary Figure S2-A The 38,534 STEC (Shiga toxin-producing *E. coli*) validation isolates were divided randomly into fifteen subsets and each subset of the isolates were combined with the identification dataset to construct a phylogenetic tree (tree 1 to 15) using Quicktree v1.3 and visualised using Grapetree’s to assign isolates in validation dataset to clusters. The representative tree (tree 1) is shown in detail as an example and all the others are similar. The scalar bar represents 0.02 substitutions per site. Known STEC clusters from identification dataset are coloured. Numbers in square brackets after cluster name are the number of isolates of each identified cluster. Isolates in validation dataset (valdb) are coloured white. An “validation” isolate was assigned to a STEC cluster if the isolate was found within a branch that exclusively contained identification dataset isolates from that cluster and that branch had a bootstrap support value of 80% or greater. ECOLI is *E. coli*. EIEC is Enteroinvasive *E. coli*. MC is STEC minor clusters.

Supplementary Figure S2-B The same phylogenetic tree as Figure S2-A was visualised using ITOL v5 which allowed bootstrap values to be displayed by colouring the internal nodes. The scalar bar represents 0.01substitutions per site. STEC (Shiga toxin-producing *E. coli*) clusters are coloured per cluster legend and shown as the ring. The internal branches are coloured to represent the bootstrap values per colour legend with green and red indicating the maximum (1) and minimum bootstrap values (0). Each cluster is supported by bootstrap value of 80% or greater. ECOLI is *E. coli*. EIEC is Enteroinvasive *E. coli*. MC is STEC minor clusters.

## Abbreviations

STEC, Shiga toxin-producing *Escherichia coli*; HC, haemorrhagic colitis; HUS, haemolytic uraemic syndrome; ESRD, end-stage renal disease; Stx, Shiga toxin; LEE, locus of enterocyte effacement; EIEC, enteroinvasive *E. coli*; MLST, multi-locus sequence typing; rSTs, ribosomal MLST STs; TP, true positives; TPR, true positive rate; TN, true negatives; TNR, true negative rate; FN, false negatives; FP, false positives.
