# Supplementary figures and images for: Improved Genomic Identification, Clustering, and Serotyping of Shiga Toxin-Producing Escherichia coli Using Cluster/Serotype-Specific Gene Markers
Source: Front Cell Infect Microbiol. 2022 Jan 10;11:772574. doi: 10.3389/fcimb.2021.772574 (PMC8785982; doi:10.3389/fcimb.2021.772574)

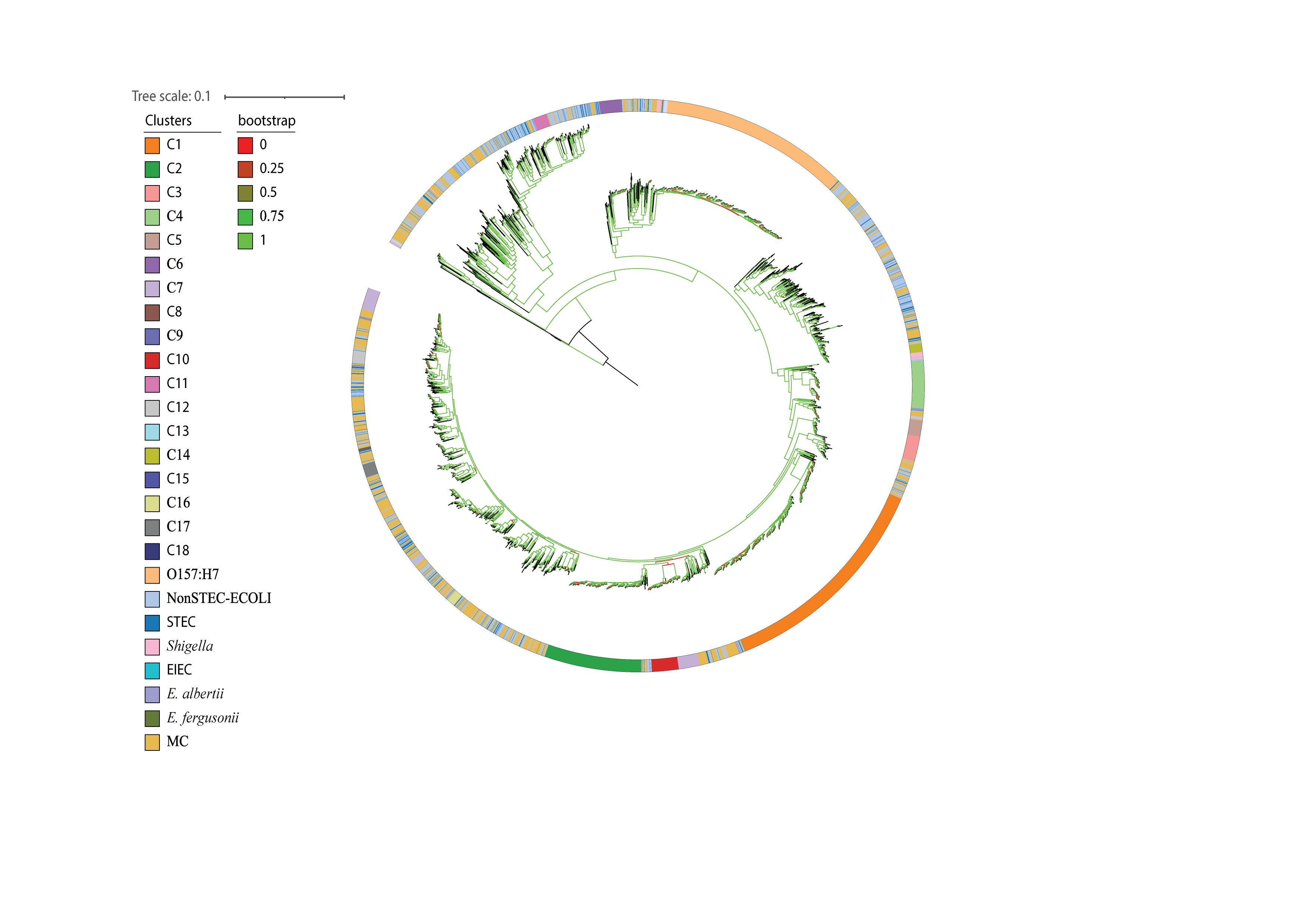

Supplement: Supplementary Figure S1 — Identification phylogenetic tree. The identification phylogenetic tree was constructed using Quicktree v1.3 as Figure 3 and was visualised using ITOL v5 which allowed bootstrap values to be displayed by colouring the internal nodes. The scalar bar represents 0.01 substitutions per site. STEC (Shiga toxin producing E. coli) clusters are colored per cluster legend and shown as the ring. The internal branches are colored to represent the bootstrap values per colour legend with green and red indicating the maximum (1) and minimum bootstrap values (0). Each cluster is supported by bootstrap value of 80% or greater. ECOLI is E. coli. EIEC is Enteroinvasive E. coli. MC is STEC minor clusters. [file Image_1.tif]

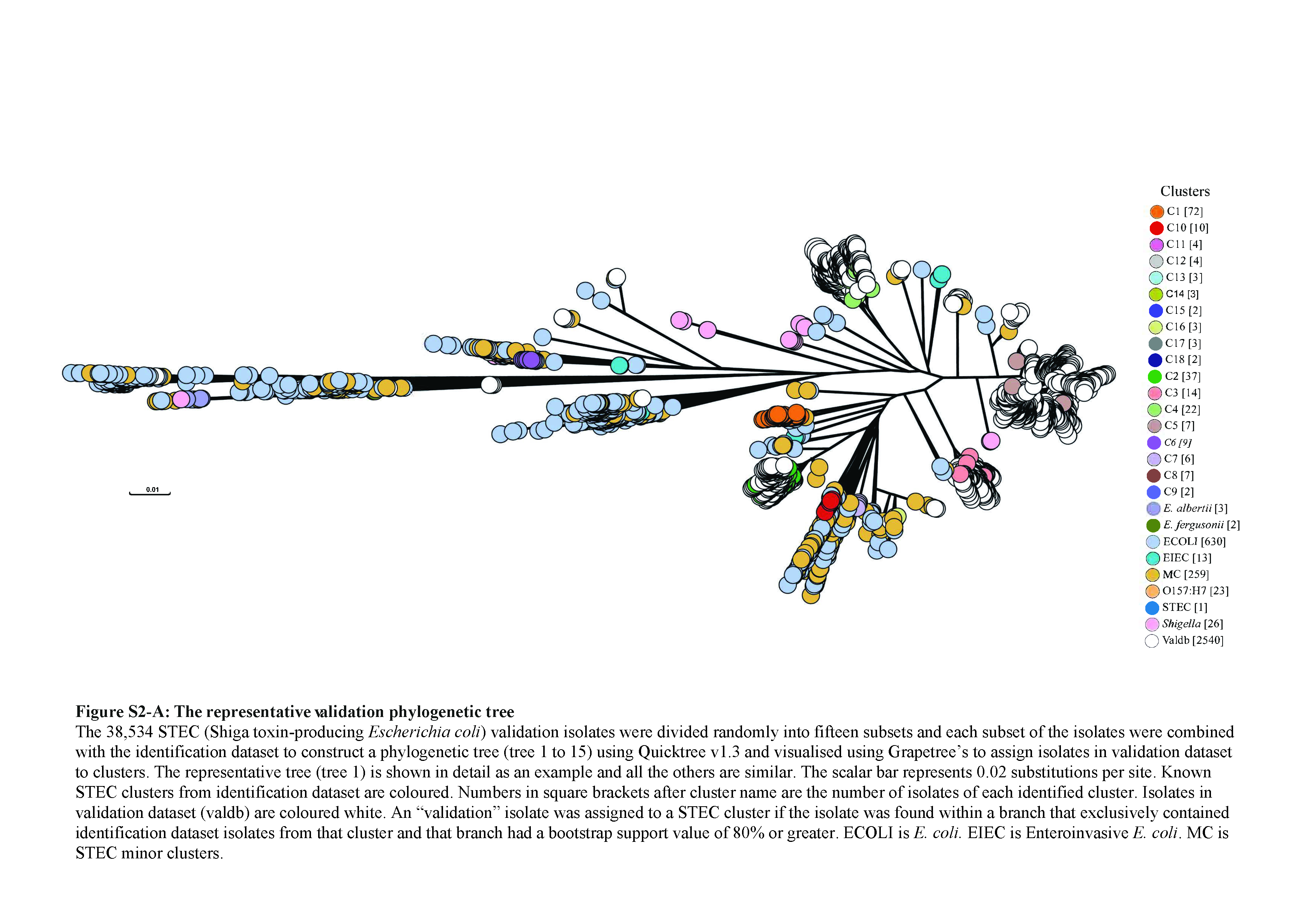

Supplement: Supplementary Figure S2 — The representative validation phylogenetic tree. [file Image_2.tiff]

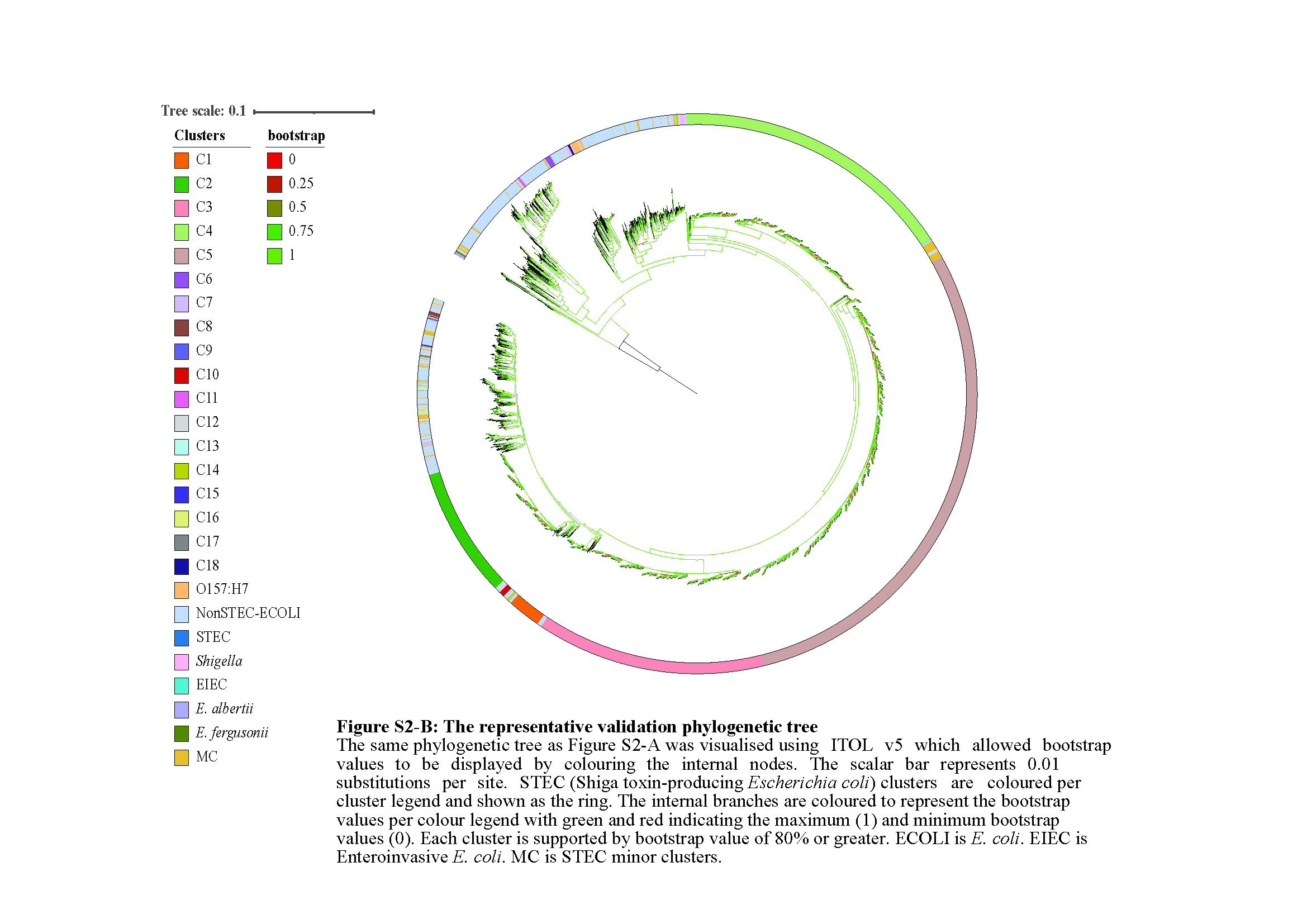

Supplement: Supplementary Figure S2-B — The same phylogenetic tree as Figure S2-A was visualised using ITOL v5 which allowed bootstrap values to be displayed by colouring the internal nodes. The scalar bar represents 0.01substitutions per site. STEC (Shiga toxin-producing E. coli) clusters are coloured per cluster legend and shown as the ring. The internal branches are coloured to represent the bootstrap values per colour legend with green and red indicating the maximum (1) and minimum bootstrap values (0). Each cluster is supported by bootstrap value of 80% or greater. ECOLI is E. coli. EIEC is Enteroinvasive E. coli. MC is STEC minor clusters. [file Image_3.tiff]
